# Apelin expression deficiency in mice contributes to vascular stiffening by extracellular matrix remodeling of the aortic wall

**DOI:** 10.1038/s41598-021-01735-z

**Published:** 2021-11-15

**Authors:** Beatrice Romier, Cédric Dray, Laetitia Vanalderwiert, Amandine Wahart, Thinhinane Hocine, Alizée Dortignac, Christian Garbar, Corinne Garbar, Camille Boulagnon, Nicole Bouland, Pascal Maurice, Amar Bennasroune, Hervé Sartelet, Laurent Martiny, Laurent Duca, Philippe Valet, Sébastien Blaise

**Affiliations:** 1 UMR CNRS 7369 MEDyC, University of Reims Champagne-Ardenne, UFR Sciences Exactes et Naturelles, Moulin de la Housse, BP 1039, 51687 Reims Cedex 2, France; 2 Institut RESTORE, Université de Toulouse, CNRS U-5070, EFS, ENVT, INSERM U1301, Université Paul Sabatier, 4bis Ave Hubert Curien, 31100 Toulouse, France; 3 Department of Biopathology, Jean Godinot Institut, Regional Cancer Control Center, Reims, France; 4 Laboratory of Biopathology, University Hospital Center, Reims, France; 5 Laboratory of Anatomy and Pathologies, Faculty of Medicine, University of Reims Champagne-Ardenne, Reims, France

**Keywords:** Physiology, Cardiology, Diseases, Endocrinology, Pathogenesis, Risk factors

## Abstract

Numerous recent studies have shown that in the continuum of cardiovascular diseases, the measurement of arterial stiffness has powerful predictive value in cardiovascular risk and mortality and that this value is independent of other conventional risk factors, such as age, cholesterol levels, diabetes, smoking, or average blood pressure. Vascular stiffening is often the main cause of arterial hypertension (AHT), which is common in the presence of obesity. However, the mechanisms leading to vascular stiffening, as well as preventive factors, remain unclear. The aim of the present study was to investigate the consequences of apelin deficiency on the vascular stiffening and wall remodeling of aorta in mice. This factor freed by visceral adipose tissue, is known for its homeostasic role in lipid and vascular metabolisms, or again in inflammation. We compared the level of metabolic markers, inflammation of white adipose tissue (WAT), and aortic wall remodeling from functional and structural approaches in apelin-deficient and wild-type (WT) mice. Apelin-deficient mice were generated by knockout of the apelin gene (APL-KO). From 8 mice by groups, aortic stiffness was analyzed by pulse wave velocity measurements and by characterizations of collagen and elastic fibers. Mann–Whitney statistical test determined the significant data (p < 5%) between groups. The APL-KO mice developed inflammation, which was associated with significant remodeling of visceral WAT, such as neutrophil elastase and cathepsin S expressions. In vitro, cathepsin S activity was detected in conditioned medium prepared from adipose tissue of the APL-KO mice, and cathepsin S activity induced high fragmentations of elastic fiber of wild-type aorta, suggesting that the WAT secretome could play a major role in vascular stiffening. In vivo, remodeling of the extracellular matrix (ECM), such as collagen accumulation and elastolysis, was observed in the aortic walls of the APL-KO mice, with the latter associated with high cathepsin S activity. In addition, pulse wave velocity (PWV) and AHT were increased in the APL-KO mice. The latter could explain aortic wall remodeling in the APL-KO mice. The absence of apelin expression, particularly in WAT, modified the adipocyte secretome and facilitated remodeling of the ECM of the aortic wall. Thus, elastolysis of elastic fibers and collagen accumulation contributed to vascular stiffening and AHT. Therefore, apelin expression could be a major element to preserve vascular homeostasis.

## Introduction

Obesity is one of the most common metabolic disorders worldwide and affects 800 million adults, 124 million children aged 5–19 years, and 58 million children aged < 5 years. The World Health Organization estimates that 2.8 million obese people die each year from complications of obesity. Arterial hypertension (AHT) is a common co-morbidity associated with obesity and is a major precursor of strokes, myocardial infarctions, heart failure, and chronic renal failure^1,2^. Many studies have found an increase in the prevalence of high blood pressure in obese patients. According to the literature^3–5^, 30–40% of obese individuals are hypertensive, and the frequency of hypertension is particularly high in obese individuals aged 45 years and older. Poirier et al*.* demonstrated a possible link between excess visceral fat and increased blood pressure^6^. Several mechanisms have been proposed to explain the link between obesity and hypertension. However, uncertainties remain, and research on this subject is ongoing. Proposed mechanisms involve the sympathetic nervous system, sodium intake, vascular wall remodeling, and even secretome deregulation of adipose tissue^2,7–9^. Apelin is an adipokine secreted by adipose tissues. Apelin plays a major role in the control of homeostasis of metabolism and cardiovascular functions and may contribute to limiting the development of pathologies, such as obesity^10^, type 2 diabetes^11^, atherosclerosis^12^, and aneurysms^13^. According to the literature, apelin and its receptor, APJ, exert a direct effect on AHT by decreasing blood pressure through a nitric oxide (NO)-dependent pathway^14^. Apelin is known to be a major regulator of inflammatory factors, which induce metabolic and cardiovascular diseases^15–18^. Inflammatory factors not only modulate the physiology of smooth muscle cells and endothelial cells but also the structure and function of the extracellular matrix (ECM), which is composed of collagen and elastic fibers. Elastic fibers, which are composed of elastin (90%) and microfibrils (10%), moderate high systolic pressure at the outlet of the heart via a phenomenon called the “Windkessel” effect^19^. In other words, the elastance capacity of these fibers within the aorta limits pressure and flow fluctuations generated by the heart during the systole–diastole cardiac cycle. During systole-diastole cycle, blood pressure generated during systolic cardiac ejection induced stretching of these elastic fibers and begetting thus, an increase of aortic radius^20^. This stretching corresponds to a storage of high systolic pressure in the form of energy which is next transformed into kinetic energy by the compliance of elastic fibers, during cardiac diastole. The consequence is a reduction of vascular radius, the transformation of pulsatile blood flow of heart into a continuous flow of blood to the rest of the arterial tree. Under physiological (aging) and pathological (diabetes) conditions, overexpression of elastases, including cathepsin S and neutrophil elastase, as well as increased activity of metalloproteinases (MMPs), such as matrix metalloproteinase 9 (MMP9), including cysteine proteases (cathepsin S), serine proteases (neutrophil elastase), and metalloproteinases (MMP9), results in elastin degradation and the production of elastin-derived peptide (EDPs), which are aging markers^20^. Elastin degradation reduces the functional properties of elastic fibers, that is to say, the loss of stretching-relaxing proprieties of elastic fibers, facilitating thus aortic stiffness. Moreover, several studies (in vivo and clinical) have suggested that stiffness generated by elastic fiber alterations is a precursor of AHT^21,22^.

Based on the findings in the literature, we also hypothesized that the absence of apelin expression would induce marked remodeling of the aortic wall, in particular, elastic fibers, thereby generating vascular stiffening and hypertension. In addition, as apelin is an important modulator of the adipocyte secretome, we investigated the contribution of the adipocyte secretome to observed morphological and functional vascular alterations. To test our hypothesis, we used an apelin-deficient murine model of adipocyte hypertrophy and high blood pressure, with apelin deficiency generated by knockout of the apelin gene (APL-KO).

## Results

### Apelin deficiency impaired aortic stiffness

**Figure 1:**
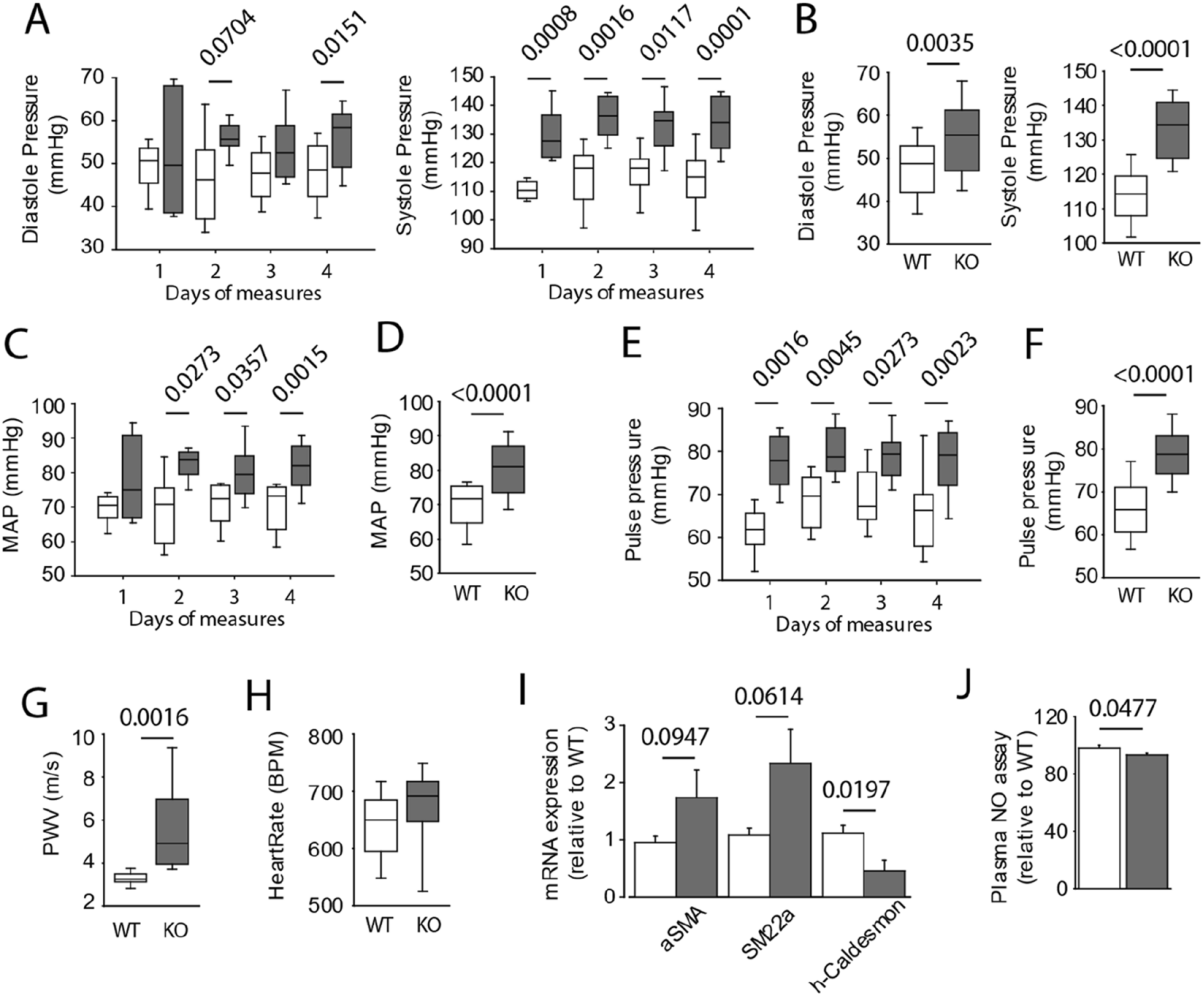
Apelin deficiency induction of arterial hypertension (AHT) in Apelin-knockout mice (KO-APL mice, grey bars, *n* = 8) as compared to wild-type mice (WT, white bar, n = 8). (**A**) Diastolic and systolic pressure measured using the tail cuff method, with all measurements obtained once per day for 4 consecutive days. (**B**) Mean arterial pressure (MAP) calculated from diastolic and systolic pressure. (**C**) Pulse pressure corresponds to the difference between diastolic and systolic pressure. (**D**) Mean pressure (systole and diastole) obtained on day 4 of the measurements (see panel **A**). (**E**) Mean MAP obtained on day 4 of the measurements (see panel **B**). (**F**) Mean pulse pressure obtained on day 4 of the measurements (see panel **C**). (**G**) Velocity of propagation of the aorta measured by the pulse wave speed (PVW). (**H**) Heart rate expressed in beat per minutes. (**I**) mRNA expression of smooth muscle contraction markers include alpha smooth muscle actin (αSMA), smooth muscle 22 alpha (SM22α), h-caldesmon. (**J**) Plasma nitrogen oxide (NO) assay. The results are the mean ± SEM. Statistically significant differences (Mann–Whitney).

As mentioned previously, apelin is an adipokine, involved in the onset of AHT. We measured arterial pressure using the “tail cuff blood pressure” method each day for 4 days (at the same time each day). The diastolic pressure values were similar in the APL-KO and WT groups (i.e., around 55 mmHg both groups) (Fig. 1A,B). In contrast, the systolic pressure (Fig. 1A,B) and mean arterial pressure (MAP) (Fig. 1C,D) were significantly elevated on all 4 days of measurements in the APL-KO mice compared to the WT mice. These results showed that the absence of apelin promotes AHT. In addition, pulse pressure, defined as the difference between the systolic and diastolic pressure, increased in KO-APL mice (Fig. 1E,F). These data might point to the development of increased vascular stiffening in the APL-KO mice. We examined aortic stiffness via pulse wave velocity (PWV) by echo Doppler (Fig. 1G). We observed a significant increase in PWV in the APL-KO mice as compared to that in the WT mice, confirming the presence of vascular stiffening in the absence of apelin expression. In order to determine whether vascular stiffening could precede or follow the presence of hypertension, we evaluated different parameters characteristic of AHT. Thus, the heart rate is not changed between the two groups (Fig. 1H). The molecular markers of smooth muscle cell contraction (Fig. 1I) such as αSMA, SM22α are not significantly increased, or even are reduced as is the h-caldesmon marker. Finally, the Mann–Whitney test showed a very slight significance as regards the decrease in the plasma level of nitrogen monoxide (NO) in the APL-KO mice (Fig. 1J). Those data may suggest that vascular stiffening could precede to AHT in our KO-APL model.

### ECM remodeling contributes to vascular stiffening in APL-KO mice

**Figure 2:**
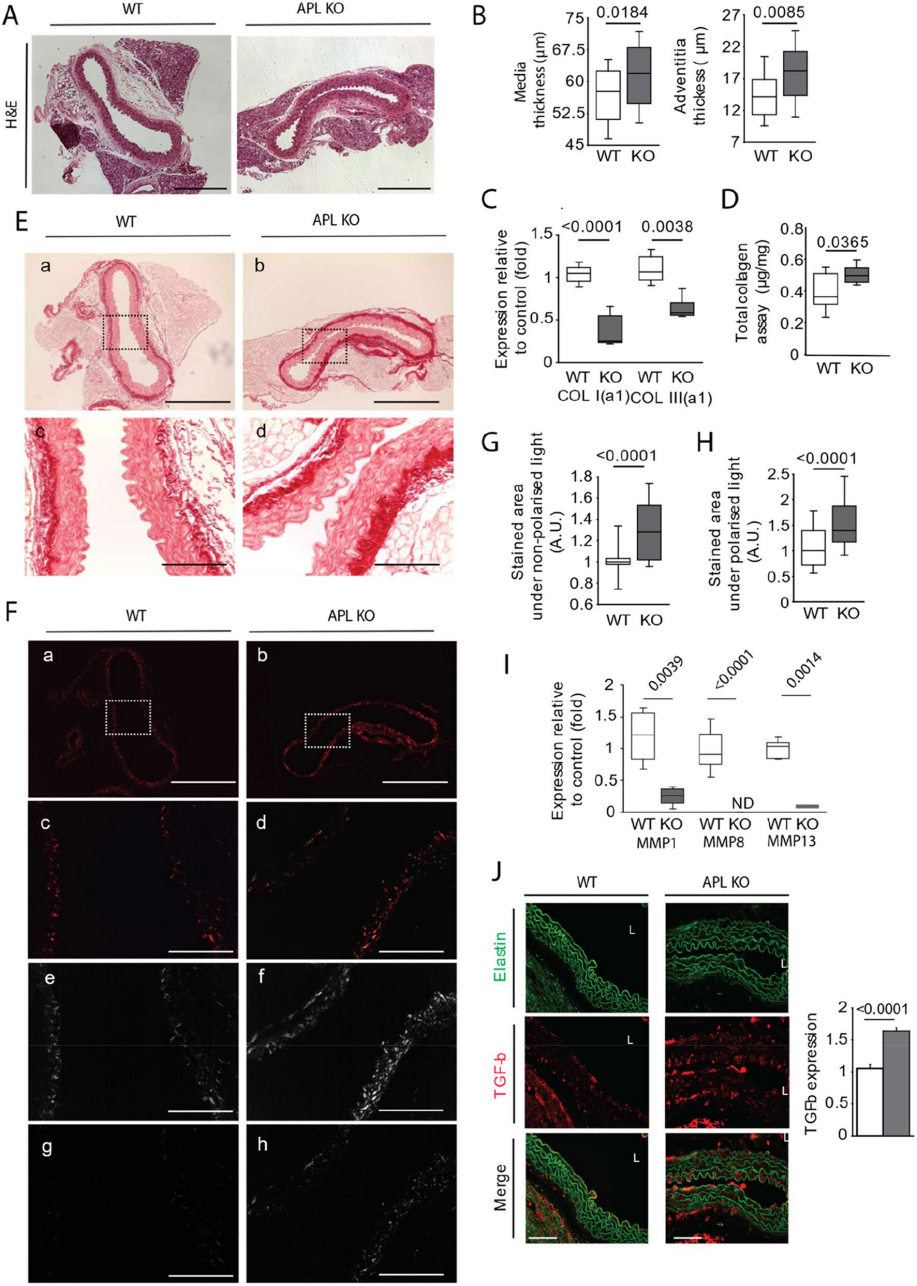
Collagen expression in the aortas of Apelin-knockout mice (KO-APL mice, grey bars, *n* = 8) and wild-type mice (WT, white bar, n = 8). (**A**) Representative hematoxylin–eosin (H&E) staining. The scale bar represents 250 μm. (**B**) Scatter plots show the intima–media tunica thickness and adventitia tunica thickness. The mean and 95% confidence interval were calculated in the normal domain after log transformation. (**C**) Total collagen assay. (**D**) mRNA expression of type I(α1) collagen and type III(α1) collagen. (**E**) Representative picrosirius red staining observed by nonpolarized light in eight slices per animal and condition (panels a and b). The scale bars for panels a–b correspond to 250 µm. Panels c and d are magnified images of panels a and b. The scale bars correspond to 60 µm. (**F**) Representative picrosirius red staining observed by polarized light (panels a and b). The scale bars correspond to 250 µm. Panels c and d are magnified images of panels a and b. The scale bars correspond to 60 µm. The scale bars for panels a–d correspond to 250 µm. The scale bars for panels e–h correspond to 60 µm. Panels e and f correspond to yellow-orange birefringence of type I collagen. Panels g and h correspond to green birefringence of type III collagen. (**G**) Semi-quantification of total collagen from picrosirius red staining visualized by nonpolarized light. (**H**) Semi-quantification of collagen I and III from picrosirius red staining visualized by polarized light. (**I**) mRNA expression of several collagenases, including metalloprotease 1 (MMP1), MMP8, and MMP13. (**J**) Immunostaining against transforming growth factor β (TGFβ), showing an TGFβ accumulation in the media in the APL-KO mice. The results are the mean ± SEM. Statistically significant differences (Mann–Whitney).

Several studies have suggested that vascular stiffening is a precursor to the onset of AHT^23–26^. We speculated that remodeling of the fibrous structures of the ECM of the aortic wall might lead to vascular stiffening. To shed light on this issue, we evaluated the thicknesses of the media tunica and adventitia tunica of the aortas of the APL-KO mice by histological staining (hematoxylin–eosin [H&E]) (Fig. 2A,B). As expected, the H&E staining revealed an increase in the thicknesses of these two layers, in accordance with other murine models of AHT. Collagen is a major component of the ECM of blood vessels and provides strength and resistance to stretching. In this study, we analyzed total collagen, type I collagen, and type III collagen expression in the aortas of the APL-KO and WT mice. Type I collagen mRNA and type III collagen mRNA were not overexpressed in the APL-KO mice as compared to that in the WT mice (Fig. 2C). However, as shown by a collagen assay (Fig. 2D) and picrosirius red staining (Fig. 2E,G), total collagen accumulation was observed in the APL-KO mice as compared to that in the WT mice. In addition, the properties of the collagen fibers in the tunica adventitia, as observed by polarized light (Fig. 2F,H), revealed an increase of orange-yellow birefringence (Fig. 2F, panels e, f) and green birefringence (Fig. 2F, panels g, h) which might be associated, respectively, to an accumulation of thick fibers, characteristic of the type I collagen on the one hand and on the other hand, to an accumulation of fine fibers, typical to type III collagen. To shed light on collagen accumulation in the APL-KO mice, we examined the expression of various collagenases, including MMP1, MMP8, and MMP13 (Fig. 2I). The results revealed significantly reduced expression of these collagenases in the aortic tissue of the APL-KO mice. According to previous studies, the reduction of collagenase expression could be explained by overexpression of transforming growth factor β (TGFβ), as observed by immunohistology (Fig. 2J). To determine whether collagen played a major role in aortic stiffness, we investigated correlations (Z-test) among systolic and pulsed pressure values, PWV, and total collagen expression (Supplementary Fig. 1A, B). The determination of total collagen and quantification by picrosirius red staining revealed no statistically significant correlation between total collagen and systolic pressure, pulsed pressure, or PWV. Thus, collagen accumulation was not a determining factor of vascular stiffening in our APL-KO murine model.

**Figure 3:**
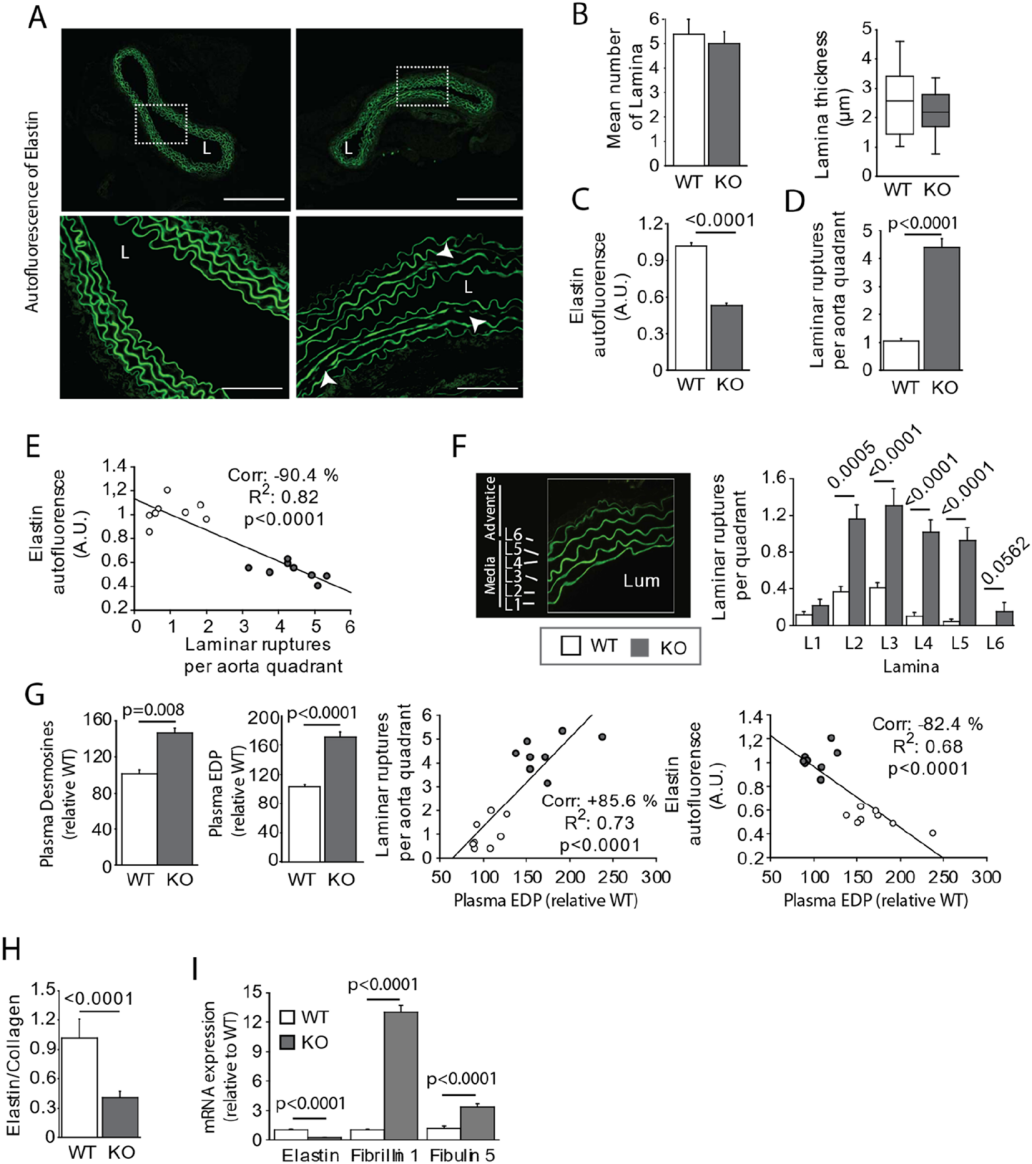
Spatial distribution of laminar ruptures in Apelin-knockout mice (KO-APL mice, grey bars, *n* = 8) and wild-type mice (WT, white bar, n = 8). (**A**) Representative autofluorescence of elastin (image of top panel). The scale bar represents 250 μm. The images in the bottom panel are magnified images of top panel. The scale bar represents 60 μm. (**B**) Bar plots indicate the number and thickness of lamina per aorta quadrant, Was something quantified according to the method of Trachet et al.^18^. (**C**) Quantification of autofluorescence level of elastin in aorta section. This quantification was performed by the staining obtained from three aorta slices were averaged per animal to account for intra-animal variation. (**D**) Laminar ruptures in four quadrants of three aorta sections per animal were calculated. (**E**) Graph depicting linear relationships between the mean elastin autofluorescence and mean laminar rupture number obtained for each animal (WT, white circles; KO, grey circles). (**F**) Representation of the numeration of elastic lamellae (L1 to L6, left panel). L, aortic lumen. Number of laminar ruptures per quadrant (right panel). (**G**) Plasma desmosine and EDP levels (left), with graphs depicting linear relationships (middle panel) between 1) plasma EDP and the mean laminar rupture number obtained for each animal studied and 2) plasma EDP and mean elastin autofluorescence obtained for each animal studied (WT, white dots; KO, grey dots). (**H**) Aortic stiffeness evaluation defined by Elastin:Collagen Ratio. Elastin correspond to elastin autofluorescence (data obtained in **C**)—collagen correspond to picrosirius red staining (data obtained in Fig. 2G). (**I**) mRNA expression of elastin, fibrillin-1, and fibulin-5 obtained by real-time qPCR (quantitative polymerase chain reaction). The results are the mean ± SEM. Statistically significant differences (Mann–Whitney).

In addition to analyze the collagen accumulation in aortic wall, we also studied the integrity of the elastic fibers. As mentioned in the introduction section, these fibers play an essential role in the phenomenon of damping of systolic pressure at outlet of the heart. Changes in the elasticity of these fibers induce vascular stiffening^27^. Figure 3A shows a representative autofluorescence image of elastin. The results of elastin revealed no significant difference in the numbers and thicknesses of the elastic fibers (Fig. 3B), although the fluorescence intensity was greatly reduced, pointing to changes in the elasticity of the fibers (Fig. 3C). Figure 3C shows many ruptures of the elastic lamellas constituting the media tunica. The rupture number was quantified by dividing the aorta into four parts (quadrants) (Fig. 3D), as described by Trachet et al*.*^18^. We noted a significant increase in the number of ruptures in the aortas of the APL-KO mice. The rupture count was inversely correlated with the decrease in the autofluorescence of elastin observed in the APL-KO mice (Fig. 3E). We also counted the number of ruptures of each elastic lamella, which we numbered 1–6, with 1 denoting the lamella closest to the tunica intima and number 6 denoting the lamella near the tunica adventitia. (Fig. 3F). Interestingly, although we observed ruptures in all the areas evaluated in the APL-KO mice, the elastic lamellae at the ends of the tunica media (lamellae 1 and 6) seemed to be the least impacted. Previous studies showed that fragmentation of elastic laminae is accompanied by the production of elastin peptides. We measured plasma EDP levels using a colorometric method and plasma desmosine levels by Elisa method (Fig. 3G), as described previously in in the literature^28^. The results revealed increased plasma EDP and desmosine levels in the APL-KO mice as compared to those in the WT mice. As shown in Fig. 3G, desmosine levels and plasma EDP levels correlated well with elastic lamella rupture. The results of autofluorescence, elastic lamella rupture count, and plasma EDP and desmosine assays all pointed to damage to the integrity of the elastic fibers in the APL-KO mice. The results of the correlation analysis between premature aging of elastic fibers, as characterized by the production of plasma EDPs and elastic lamella rupture count, and functional parameters (systolic pressure, pulsed pressure, and PWV) shows that fragmentation of elastic fibers is a determining parameter of vascular stiffening (Supplementary Fig. 1C, D).

Numerous studies reported that arterial stiffness is a consequence of an increase in collagen synthesis and a decrease in the elasticity of the media tunica. To confirm that apelin deficiency is the origin of arterial stiffness, we quantified the elastin:collagen ratio in the APL-KO mice based on autofluorescence (Fig. 3C) and the collagen ratio, with the latter determined by labeling with picrosirius red staining (Fig. 2C). As shown in Fig. 3H, the ratio of collagen was higher than that of elastin. These data explain the vascular stiffening observed in the APL-KO mice. Finally, we investigated the elastogenesis of the elastic fibers by analyzing the mRNA expression of elastin, fibrillin 1, and fibulin 5 (Fig. 3I). The qPCR results revealed increased expression of fibrillin 1 and fibulin 5 and reduced expression of elastin. As depicted in Fig. 2, TGFβ, which induces elastogenesis, was overexpressed in the APL-KO mice. Together, these findings point to dysfunction of elastic fiber formation and repair in our APL-KO murine model.

### High elastase activity in APL-KO Mice induces premature aging of elastic fibers

**Figure 4:**
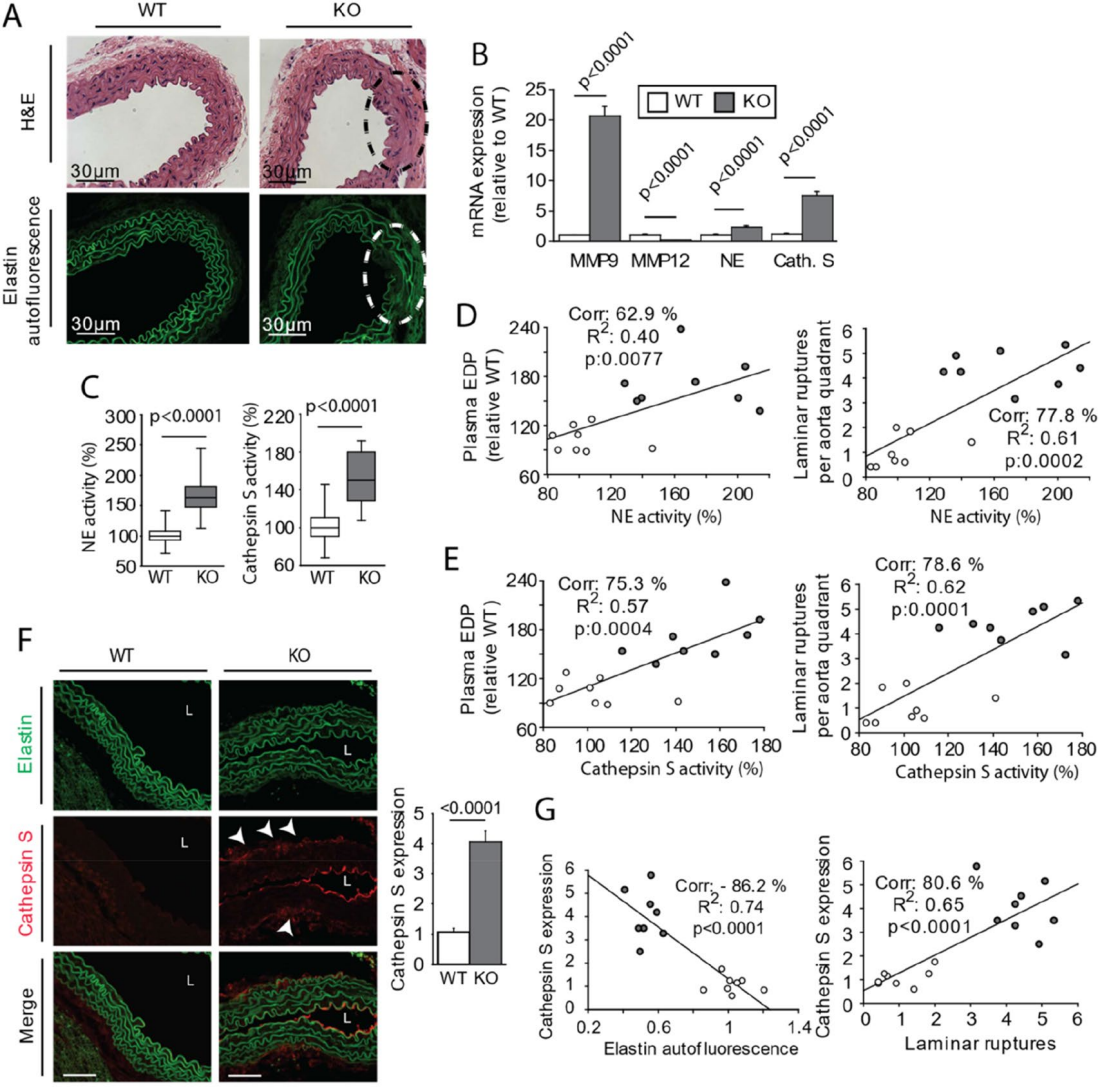
Increase of the activity of cathepsin S in the aortic wall of Apelin-knockout mice (KO-APL mice, grey bars, *n* = 8) as compared wild-type mice (WT, white bar, n = 8). (**A**) Hematoxylin–eosin (H&E) staining and autofluorescence of elastin showing fragmentation of inner laminae (L1–L4), a characteristic of aneurism development. (**B**) mRNA expression of matrix metalloproteinases (MMPs) -9 and 12. Neutrophil elastase (NE), Cathepsin S (Cath. S). (**C**) Bar plots indicate neutrophil elastase activity (left) and cathepsin S activity (right). (**D**) Graph depicting linear relationships between neutrophil elastase activity and plasma EDP levels (right) and mean laminar rupture number obtained for each animal studied (right, WT, white dots; KO, grey dots). (**E**) Graph depicting linear relationships between cathepsin S activity (left) and plasma EDP levels and the mean laminar rupture number obtained for each animal studied (right, WT, white circles; KO, grey circles). (**F**) Immunostaining against cathepsin S showing accumulation in lumen of aorta (L) near the tunica intima and between the tunica media and tunica adventitia (arrows). (**G**) Graph depicting linear relationships between cathepsin S staining and laminar rupture number (left) and elastin autofluorescence (right, WT, white circles; KO, grey circles). The results are the mean ± SEM. Statistically significant differences (Mann–Whitney).

**Figure 5:**
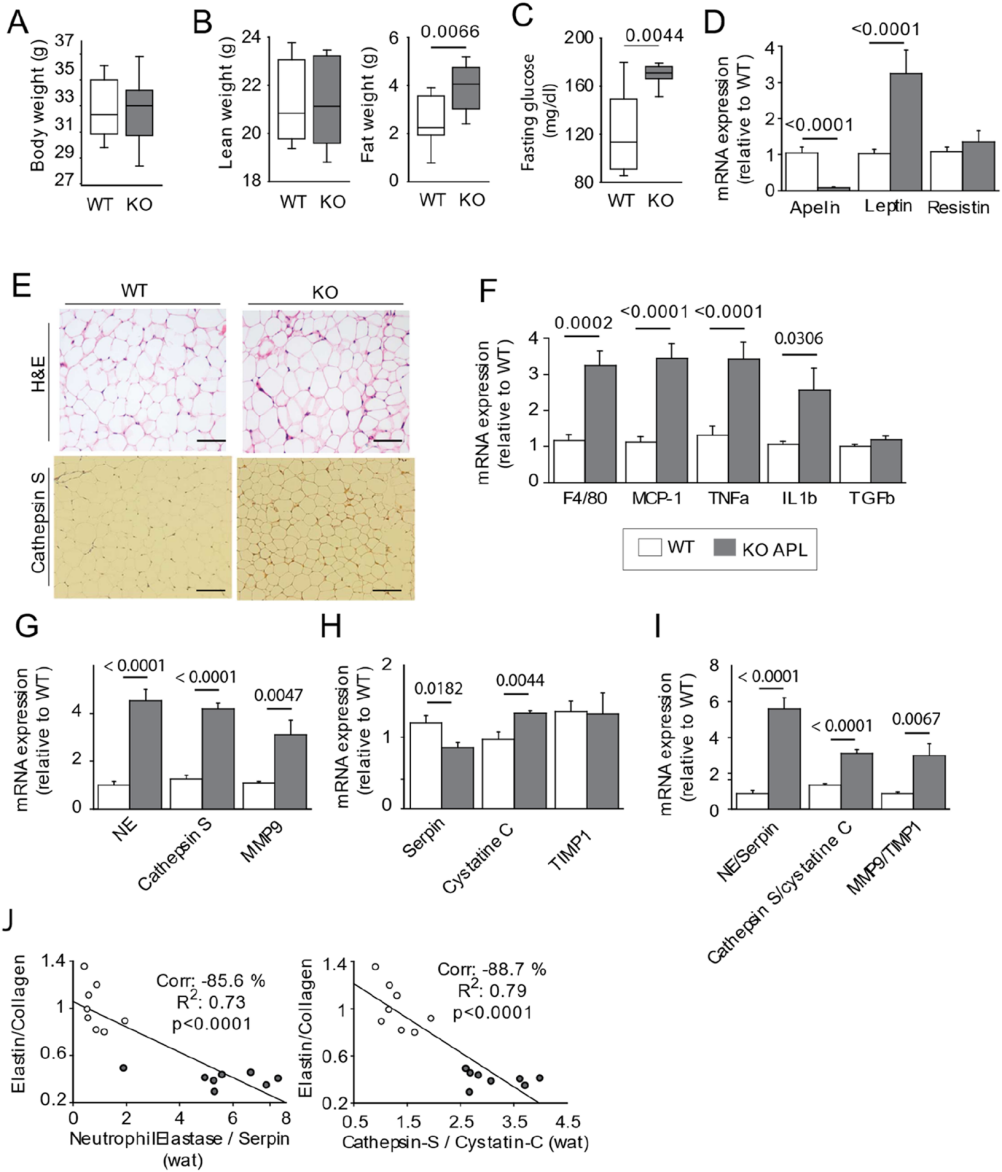
Remodeling of visceral adipose of Apelin-knockout mice (KO-APL mice, grey bars, *n* = 8) as compared wild-type mice (WT, white bar, n = 8). (**A**) Body weight, (**B**) lean and fat weights (**C**) Blood fasting glucose. (**D**) mRNA expression of leptin and resistin in perigonadic white adipose tissue. (**E**) Hematoxylin & Eosin staining (top) and immunostaining against Cathepsin S (bottom) (**F**) mRNA expression of inflammatory markers, including F4/80 and monocyte chemoattractant protein 1, tumor necrosis factor (TNFβ), interleukin 1β (IL1β), and transforming growth factor β (TGFβ). (**G**) mRNA expression of elastases, such as neutrophil elastase, cathepsin S, and MMP9 (metalloproteinase). WT, white bars; APL-KO, grey bars. (**H**) mRNA expression of Elastase inhibitors (serpin, cystatin C, and tissue inhibitor of metalloproteinase 1 [TIMP1]). (**I**) Elastases and its inhibitors Ratio. (**J**) Univariate correlation obtained by Z-test between the aortic stiffness (determined from elastin:collagen ratio and shown in Fig. 3J) and visceral adipose tissue remodeling (determined from the protease:inhibitor ratio and shown in **I**). The results are the mean ± SEM. Statistically significant differences (Mann–Whitney).

**Figure 6:**
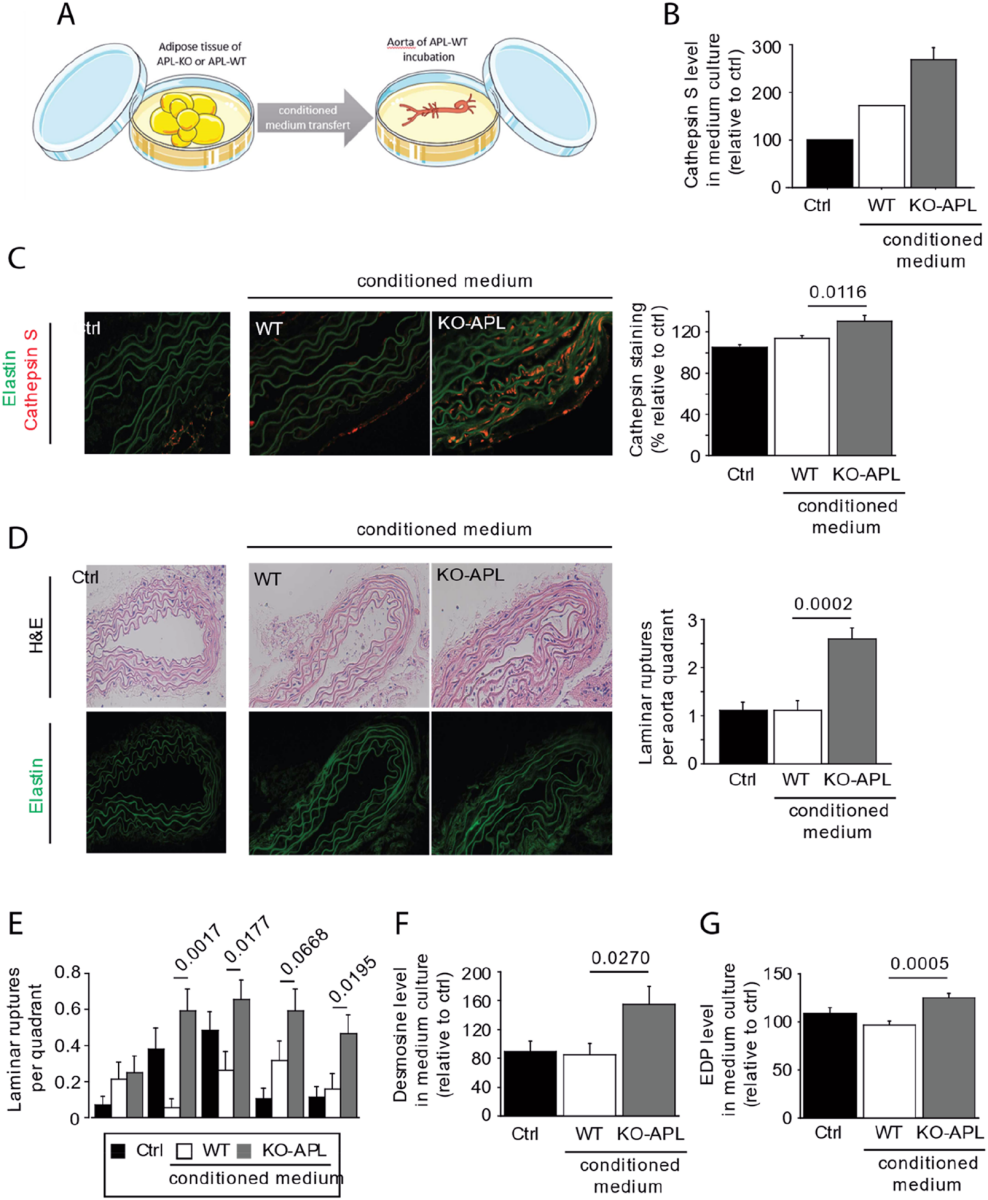
Effects of conditioned medium obtained from visceral adipose tissue of Apelin-knockout mice (KO-APL mice, grey bars, *n* = 4) and wild-type mice (WT, white bar, n = 4) on elastic fiber fragmentation of WT aorta. (**A**) Schematic of the protocol of conditioned medium, applied to the aortas of WT mice. The control condition (Crtl, black bar) corresponded to a medium that had not been in contact with adipocytes. Image was modified from Servier Medical Art, licensed under a Creative Common Attribution 3.0 Generic License. http://smart.servier.com/. (**B**) Identification of the activity of cathepsin S in the media after incubation of visceral adipocytes from APL-KO or WT mice but before the incubation with WT aorta. (**C**) Anti-cathepsin S immunostaining in the aorta after 3 days of incubation with the conditioned medium. (**D**) Histology (hematoxylin–eosin [H&E]) and autofluorescence of aorta elastin after 3 days of incubation with the conditioned medium. (**E**) Number of laminar ruptures per quadrant after 3 days of incubation with the conditioned medium. (**F**) Desmosine level found in culture medium, after 3 days of incubation with the conditioned medium (**G**) Elastin-derived peptide (EDP) level found in culture medium, after 3 days of incubation with the conditioned medium.

We observed that 25% of the mice in the APL-KO group, presented a high lamella rupture count in the inner laminae of the aorta (Fig. 4A), suggesting an high activity of elastases, coming from blood circulation or cells bordering lumen of aorta. Previous studies reported that this type of elastin fragmentation is induced by overexpression of elastases. Although MMP12 expression was significantly reduced (Fig. 4B), the expression of proteases, including MMP9, neutrophil elastase, and cathepsin S, was markedly increased. This result pointed out significant proteolytic activity in the blood vessel. Next, we measured the plasma activity of neutrophil elastase and cathepsin S (Fig. 4C) and found that the activities of both were significantly increased in the APL-KO mice. As reported in the literature, these activities are significantly correlated with elastic lamella fragmentation, as well as with the production of EDPs (Fig. 4D,E). Immunostaining confirmed the presence of cathepsin S around elastic lamellae (Fig. 4F) associated to elastin fragmentation (Fig. 4G). Cathepsin S was present at tunica media and in the lumen of the aorta near the tunica intima suggesting that cathepsin S is transported in blood. Cathepsin S is secreted by smooth muscle cells, as well as by inflammatory cells, such as macrophages. Obesity and insulin resistance are chronic inflammatory diseases of adipose tissue. In this study, we used EchoMRI to shed light on remodeling of visceral adipose tissue. As shown by the results, although weight (Fig. 5A) and lean mass (Fig. 5B) were unchanged in the APL-KO mice, visceral fat pad (Fig. 5B) developed in the APL-KO mice in association with an increase of fasting glucose (Fig. 5C) and mRNA overexpression of adipokines, such as leptin (Fig. 5D). The results of H&E staining showed that the sizes of lipid droplets increased in the APL-KO mice (Fig. 5E), with this increase associated with an increase in inflammatory markers, such as macrophages (monocyte chemoattractant protein 1 and F4/80), and cytokines, including tumor necrosis factor β (TNFβ) and interleukin β (ILβ). TGFβ was unaffected by KO of apelin expression (Fig. 5F). In several tissues, inflammation is associated with elevated expression of proteases. As shown in Fig. 5G, cathepsin S, neutrophil elastase, and MMP9 expression were significantly increased in the APL-KO mice. In addition, apelin deficiency influenced the expression of their inhibitors, cystatin C, serpin, and tissue inhibitors of metalloproteinase 1 (TIMP-1), respectively, as shown in Fig. 5H. Nevertheless, the ratios between protease expression and its inhibitor show that the balance is in favor to protease activity (Fig. 5I). These results confirmed that KO of apelin regulate lipid metabolism, inflammation, and protease expression. These data suggest that apelin expression plays a role in the activation of elastases and lipogenesis, which may lead to vascular complications. To determine whether cathepsin S produced in visceral adipose tissue could influence the aortic stiffness, we performed a correlation Z-test, between the elastin/collagen ratio, mentioned in Fig. 3H and elastases of visceral adipose tissue descripted in Fig. 5I. The negative correlation between those last factors suggests that cathepsin S or neutrophil elastase produced during remodeling of fatty tissue may contribute to premature aging of elastic fibers (Fig. 5J). To shed light on the adipocyte secretome, visceral adipose tissue from the APL-KO and WT mice were incubated in culture medium. Aortic tissue from the WT mice was then incubated with this conditioned medium or not for 3 days (Fig. 6A). Before incubation with the aorta, we measured the level of Cathepsin S and neutrophile elastase activities in conditioned medium. While neutrophile elastase activity was not detected, the activity of cathepsin S in the adipose tissue of the conditioned medium increased in the APL-KO condition as compared to that in the WT condition. (Fig. 6B). H&E staining and elastin autofluorescence (Fig. 6C) after 3 days of incubation with the conditioned medium revealed a significant increase in ruptures of elastic fibers of the aorta incubated with the conditioned medium APL-KO (Fig. 6D,E). Elastin fiber fragmentation was associated with an increase in the production of desmosine (Fig. 6F) and EDP levels (Fig. 6G) in the medium. These data suggested that cathepsin S secreted by the secretome of the adipose tissue of the APL-KO mice is capable of inducing fragmentation of elastic lamellae of the aorta.

## Discussion

We demonstrated for the first time in this study that the absence of apelin expression in mice leads to an increase in vascular stiffening. This stiffness was due to significant remodeling of the ECM. In this study, we demonstrated collagen accumulation, in particular type I and type III collagen, in the APL-KO mice. Collagen accumulation in the tunica adventitia was due to a decrease in the expression of collagenases, such as MMP1, -8, and -13. Collagen accumulation changes the biomechanical properties of vessels^29^. Structural rigidity of collagen fibers limits the initial capacity for elastance and compliance in response to variations in cardiac pressure, in according to the laws of Hooke and Laplace^20^. Other than the collagen accumulation, the elasticity properties of the aorta are dependent on the integrity of the elastic fibers^20^. In APL-KO mice, we demonstrated increased fragmentation of elastic fibers, with parallel high production of EDPs. The production of EDPs is descripted as an aging marker, in particularly of elastin, the main constituent of those fibers^30^. Premature aging of fibers in APL-KO mice, limits the elasticity capacity of the aorta, further promoting vascular stiffening. As reported in the literature, from the period of adolescence onward, no new functional elastic fibers can be produced and altered elastic fibers cannot be repaired^31^. In our study, significant upregulation of the expression of fibrillin-1 and fibulin 5 while downregulation of elastin expression are observed in the APL-KO mice. Upregulation of fibrillin-1 and fibulin expressions may be an adaptative compensatory response to the loss of elasticity. Those overexpressions could be induced by TGFβ, which is a major agent of elastogenesis^32^. TGFβ is known to play a role in collagen accumulation by downregulation of the expression of collagenases, such as MMP1, -8, and -13^33,34^. Collagen accumulation, on the one hand, and loss of elastic fiber integrity, on the other hand, are two potential anatomical explanations for the increase in PWV observed in the APL-KO mice in the present study.

In the APL-KO model, we observed both vascular stiffening and AHT. A previous study on AHT reported that the apelin receptor APJ stimulated in endothelial cells, the production of NO, a powerful vasorelaxant of smooth muscle cells^14^. We cannot exclude this possibility in arteries of small caliber in our model, although the effects of apelin/APJ would be less apparent in elastic arteries, such as the aorta. We speculate that hypertension could also be due to aortic stiffness and loss of elastic fiber functionality. Indeed, the integrity of elastic fibers seems to strongly influence blood pressure. In single-allele mouse models for the elastin gene (ELN+/−), arterial pressure increased by approximately 25% in both young and old mice^21,22^. Likewise, clinical studies involving cohorts of patients with genetic elastin deficits (patients with supravalvular aortic stenosis or Willliams–Beuren syndrome) presented not only with vascular stiffening but also with AHT^35,36^. These murine and clinical models suggested that the absence of elastin and therefore of functional elastic fibers was the cause of hypertension in these individuals. In addition, there is a large body of recent data indicating that hypertension is a measurable symptom of underlying cardiovascular disease, and that an increase in arterial stiffness precedes hypertension^23–26^. These same studies suggest that vascular stiffening is a more sensitive indicator of future cardiovascular diseases than are arterial pressure measurements. In our APL-KO model, we were able to study the continuum of cardiovascular disease in our model from vascular stiffening to the development of hypertension. An increase in systolic pressure, which is a characteristic of hypertension, can exert a significant biomechanical force against the aortic walls^20^. This can contribute to acceleration of elastic fiber fragmentation, increased stiffness, and an increased risk of rupture of the arterial wall.

Finally, in the present study, we also sought to determine the factors that could explain premature aging of elastic fibers and thus explain the occurrence of vascular stiffening. During physiological aging, the main cause of elastic fiber wear is mechanical constraints of blood aorta. Indeed, during an individual’s lifetime, elastic fibers oscillate 2 billion times between relaxed and extended conformations^20^. Over time, fragmentation of elastic fibers is inevitable. In addition to these mechanical constraints, there are enzymatic constraints due to the overexpression of proteases. Although the phenomenon of elastolysis is not yet fully understood, several elastases, including cysteine proteases (cathepsin S), serine proteases (neutrophil elastase), and metalloproteases (MMP9), have been identified^30^. The activity of these enzymes is dependent on inflammation. As obesity and insulin resistance are by definition chronic inflammatory diseases, the expression of these elastases is increased in the presence of both obesity and insulin resistance. In this context, an increase in the expression of proteases, such as cathepsin S, has been described as a marker of the degree of obesity^37,38^. In our APL-KO mouse model, we demonstrated that proteases, including cathepsin S, were widely expressed not only in adipose tissue but also in vascular tissue. The activity of these proteases is dependent on the level of expression of their inhibitors, serpin (inhibitor of neutrophil elastase), cystatin C (inhibitor of cathepsin S), and TIMP-1 (an inhibitor of MMP9)^39,40^. In the APL-KO mice, elevated activity of elastases was associated with elastic fiber fragmentation as well as increased production of EDPs. These observations clearly confirmed that the absence of apelin is synonymous with accelerated aging of the aorta. Elastases, such as cathepsin S, can be synthesized by smooth muscle cells in the tunica media or by fibroblasts in the tunica adventitia. In the present study, we investigated whether the increase in elastase activity/expression due to increased visceral adiposity contributed to elastolysis in the APL-KO mice, as suggested by the correlation analysis. Using conditioned medium prepared from visceral adipocytes obtained from APL-KO mice, we demonstrated increased fragmentation of elastic fibers of aortas obtained from the WT mice. Thus, the absence of apelin modified the secretome of adipocytes by promoting, for example, the release of leptin or cathepsin S. Leptin is described in the literature as a hypertensive factor. Nevertheless, studies also note that the expression of proteases, such as MMP2^41^, a precursor of MMP9 activity or cathepsin S^42^, inhibits the effects of leptin, including, maybe, hypertensive effects. Based on what findings in the present study, we believe that the activities of elastases, such as cathepsin S, were mainly responsible for the vascular stiffening and hypertension observed in the APL-KO mice^43,44^. In this study, we show for the first time that the overexpression of inflammation-related proteases during obesity induces major macrovascular effects.

In conclusion, the absence of apelin expression induces marked metabolic and inflammatory disturbances characteristic of obesity. Changes within adipocyte tissue, in particular increased activity of cathepsin S, contributes to major remodeling of the ECM of the aortic wall. This remodeling is associated with an increase in aortic stiffness and the onset of AHT. Therefore, in the context of obesity, apelin expression seems essential to preserve vascular homeostasis.

## Methods

### Animals

All animal experiments were performed in compliance with the guidelines of the French Institute of Medical Research (INSERM) and were approved by the animal ethics committee of Toulouse University on the use and care of animals for animal research. The study complied with ARRIVE guidelines (Animal Research: Reporting of In Vivo Experiments). APL-KO mice were generated by homologous recombination of a targeting vector in embryonic stem cells (Genoway, Lyon, France)^45^. Recombined embryonic stem cell clones were injected into C57BL/6J-derived blastocysts to generate chimeric mice. Constitutive KO heterozygous females and homozygous males were characterized by Polymerase chain reaction (PCR) and a Southern blot, Twelve-week-old APL-KO and WT female mice (*n* = 8/groups) were maintained in cages under a 12:12 h light/dark cycle in a temperature- and humidity-controlled environment. This choose of sex was based on the fact that 'at the same age, women have more cardiovascular risk factors than men, in particularly for vascular stiffness^23,46,47^. The animals had access to a normal diet (AIN-93M rodent diet, Special Diet Service, Witham, U.K.) and water ad libitum during the experimental period.

### Plasma assays

Evaluations of EDP concentrations were performed using a commercially available kit (Biocolor, Antrim, U.K.) according to Blaise et al.^48^ or a desmosine ELISA kit (Cusabio Technology LLC, Houston, TX, U.S.) according to Balin et al*.*^28^. A neutrophil elastase activity assay and cathepsin activity assay (Abcam, Cambridge, U.K.) were used to measure neutrophil elastase and cathepsin S activities, respectively, according to Romier et al.^49^ and Balint et al.^28^.

### Blood pressure measurement using the tail cuff method

Systolic blood pressure was monitored using the tail cuff method, with the aid of a computerized system (BP 2000 Blood Pressure Analysis System; Visitech Systems, Apex, NC, U.S.). Acclimatization of mice was performed the week before the final blood pressure measurements. Blood pressure was measured once per day for 4 consecutive days. Each day, 10 measurements were obtained from each animal, and the average was recorded. If the systolic blood pressure measurements of the APL-KO mice were higher than those of the control mice (WT mice) on each of the 4 days, the mice were considered to be hypertensive.

### Pulse wave velocity (PWV) assay

Doppler ultrasound (Indus Doppler Flow Velocity; Webster, TX, U.S.) was performed under gas anesthesia with 2% isoflurane (Centravet, Nancy, France). The mice were placed supine on a heating board, with electrocardiographic electrodes fixes to their legs. The Doppler probes were placed on the transverse aortic arch (10 MHz) and abdominal aorta (20 MHz), and the distance between the probes was determined using a precision caliper. The pre-ejection time and the time between the R-wave of the electrocardiogram and the base of the Doppler signal was determined, using at least eight signals for each measure. Aortic PWV was calculated by dividing the distance (cm) between the probes by the difference in pre-ejection times (milliseconds) of the thoracic and abdominal regions.

### Composition of body fat mass

To determine fat and lean mass, the mice were placed in a clear plastic holder without anesthesia or sedation. Fat and lean mass were then determined using an EchoMRI-3-in-1 system (Echo Medical Systems, Houston, TX, USA).

### Histology

The aorta samples were embedded in paraffin. Three transversal aorta sections were stained with H&E or picrosirius as described by Pooya et al.^50^. Picrosirius red staining was observed by microscopy under polarized light (Leica, Paris, France). Picrosirius red staining and the thicknesses of the tunica media and tunica adventitia were quantified from three different sections per animal using ImageJ software (*n* = 16). The expression of cellular and ECM proteins were assessed by immunolabeling the aortic tissue sections (4 µm) with primary antibodies targeting cathepsin S or TGFβ (Santa Cruz Biotechnology, Dallas, TX U.S.). Image acquisitions were achieved through microscopy (Olympus, Paris, France) and analyzed by ImageJ software. Elastin autofluorescence in tissue sections was recorded with excitation at 488 nm according to the method of Romier et al.^49^. Each staining was quantified according to the method of Trachet et al.^18^. Lamellar rupture and thickness and fiber number and elastin autofluorescence were quantified using ImageJ software.

### Gene expression analysis

Gene expression was analyzed by a qPCR test as previously described. Briefly, total RNA was extracted using Trizol reagent (Eurobio Scientific, Les Ulis, France). The RNA concentration was measured using a NanoDrop system (ThermoFisher Scientific, Illkirch, France). The 260/280 ratio was calculated using NanoDrop software and used to evaluate protein contamination. Complementary DNA (cDNA) was generated using a Verso cDNA kit (ThermoScientific, Illkirch, France). Real-time PCR was performed using SYBR Green on a BioRad CFX96 Real Time System (BioRad, Hercule, Californie, U.S.). In this study, 5 µl of cDNA (1/10) and 0.7 µl of each forward and reverse primers (3 µM) were used for the qPCR test, with cycling conditions as follows: 95 °C for 15 min, 40 cycles of 95 °C for 10 s and 60 °C for 60 s. RNA expression was normalized to the housekeeping genes 36B4 and RPS26, and relative gene expression was calculated using the 2 − ΔΔCT method. The sequences for the qPCR primers are listed in Table 1.

**Table 1:** Sequences used for qPCR.

Gene name	Forward sequence (5′ → 3′)	Reverse sequence (5′ → 3′)
RPS26	TAGAAGCCGCTGCTGTCAGG	GGCACAGCTCACGCAATAATG
36B4	AAAGCCTGGAAGAAGGAGGTC	AGATTCGGGATATGCTGTTGG
MMP-9	CACGGAGACGGGTATCCCTT	GGGCACCATTTGAGTTTCCAT
Neutrophil elastase	TGGAGGTCATTTCTGTGGTG	CTGCACTGACCGGAAATTTAG
Elastin	GCTGCTGCTAAGGCTGCTAA	AGCACCTGGGAGCCTAACTC
Fibulin 5	ATCTGCTGATTGGTGAAAACC	ATGGTGAATGGCTGGTCTCT
Fibrillin 1	GGACGGAAAGAACTGTGAAGAT	ACACATTCCGTTTAGGCACA
Cathepsin S	GCGTCACTGAGGTGAAATACC	CCCCCACAGCACTGAAAG
Cystatin C	ATGACCAGCCCCATCTGA	CCAGGGCACGCTGTAGAT
Serine peptidase inhibitor	TAGGGAGCAAGGGTGACACTC	ACTGTCTGGTCTGTTGAGGGT
Timp1	TCCCCAGAAATCAACGAGACC	GTACCGGATATCTGCGGCATT
F4/80	CTTTGGCTATGGGCTAGTC	GCAAGGACAGAGTTTATCGTG
Tnf	TGGGACAGTGACCTGGACTGT	TTCGGAAAGCCCATTTGAGT
Interleukin 1 beta	CAGGCAGGCAGTATCACTCA	GTCACAGAGGATGGGCTCTT
Tgfb 1	TGGAGCAACATGTGGAACTC	GTCAGCAGCCGGTTACCA
Leptin	GACACCAAAACCCTCAT	CAGTGTCTGGTCCATCT
Resistin	TTCCTTGTCCCTGAACTGCTG	GCTGGAAACCACGCTCACTT
Mmp12	TTGTGGATAAACACTACTGGAGGT	AAATCAGCTTGGGGTAAGCA
Actin alpha 2, smooth muscle, aorta	TCCACGAAACCACCTATAACAG	CAGAGTACTTGCGTTCTGGAG
SM22 alpha	CCCAGACACCGAAGCTACTC	GACTGCACTTCTCGGCTCAT
h-caldesmon	TACACCAATGCAATCGAGGGAA	TACATCTCCTGGCCTCAAGTCA
MMP-1	CACTCCCTTGGGCTCACTCA	GTTGCACCTGTTGGCTGGAT
MMP-8	AACGGGAAGACATAC	GGGTCCATGGATCTT
MMP-13	CAGTCTCCGAGGAGAAACTATGAT	GGACTTTGTCAAAAAGAGCTCAG

### Conditioned medium preparation

Perigonadal white adipose tissue (visceral tissue, 250 mg) were taken from the WT and APL-KO mice and dissected into small cubes (5 mm wide) and rinsed in according with Boucher et al.^51^. These cubes were then incubated in Dulbecco's Modified Eagle's Medium (DMEM) F12 (Lonza, Colmar, France) with 1% bovine serum albumin, with light agitation. The medium was removed after 24 h of incubation. The thoracic aortas of the WT mice (*n* = 4/medium) were then removed and incubated with the conditioned culture media (APL-KO or WT) or with normal DMEM F12 with 1% bovine serum albumin (i.e., had not been in contact with fatty tissue). This last condition served as a control for the experiments.

### Statistical analysis

Statistical analyses were conducted using Statview software. Comparisons between the APL-KO and WT mice (*n* = 8 in both groups) are presented as mean ± standard error of the mean (SEM Comparisons among normal distributions were made by a *t*-test and among non-normal distributions by a Mann–Whitney *U* test). Univariate correlation analysis (Z-test) was conducted to determine the relationship between two parameters. A value of *P* < 0.05 was considered to indicate significance and in each figure, we indicated the actual p-value.

## Supplementary Information

Supplementary Figure 1.
