# Supplementary material for: Apelin expression deficiency in mice contributes to vascular stiffening by extracellular matrix remodeling of the aortic wall
Source: Sci Rep. 2021 Nov 15;11:22278. doi: 10.1038/s41598-021-01735-z (PMC8593139; doi:10.1038/s41598-021-01735-z)
Supplement: Supplementary file 1 — Supplementary Figure 1. [file 41598_2021_1735_MOESM1_ESM.pdf]

Apelin expression deficiency in mice contributes to vascular stiffening by extracellular matrix remodeling of the aortic wall

Beatrice Romier, Cédric Dray, Laetitia Vanalderwiert, Amandine Wahart, Thinhinane Hocine, Alizée Dortignac, Christian Garbar, Corinne Garbar, Camille Boulagnon, Nicole Bouland, Pascal Maurice, Amar Bennasroune, Hervé Sartelet, Laurent Martiny, Laurent Duca, Philippe Valet, Sébastien Blaise

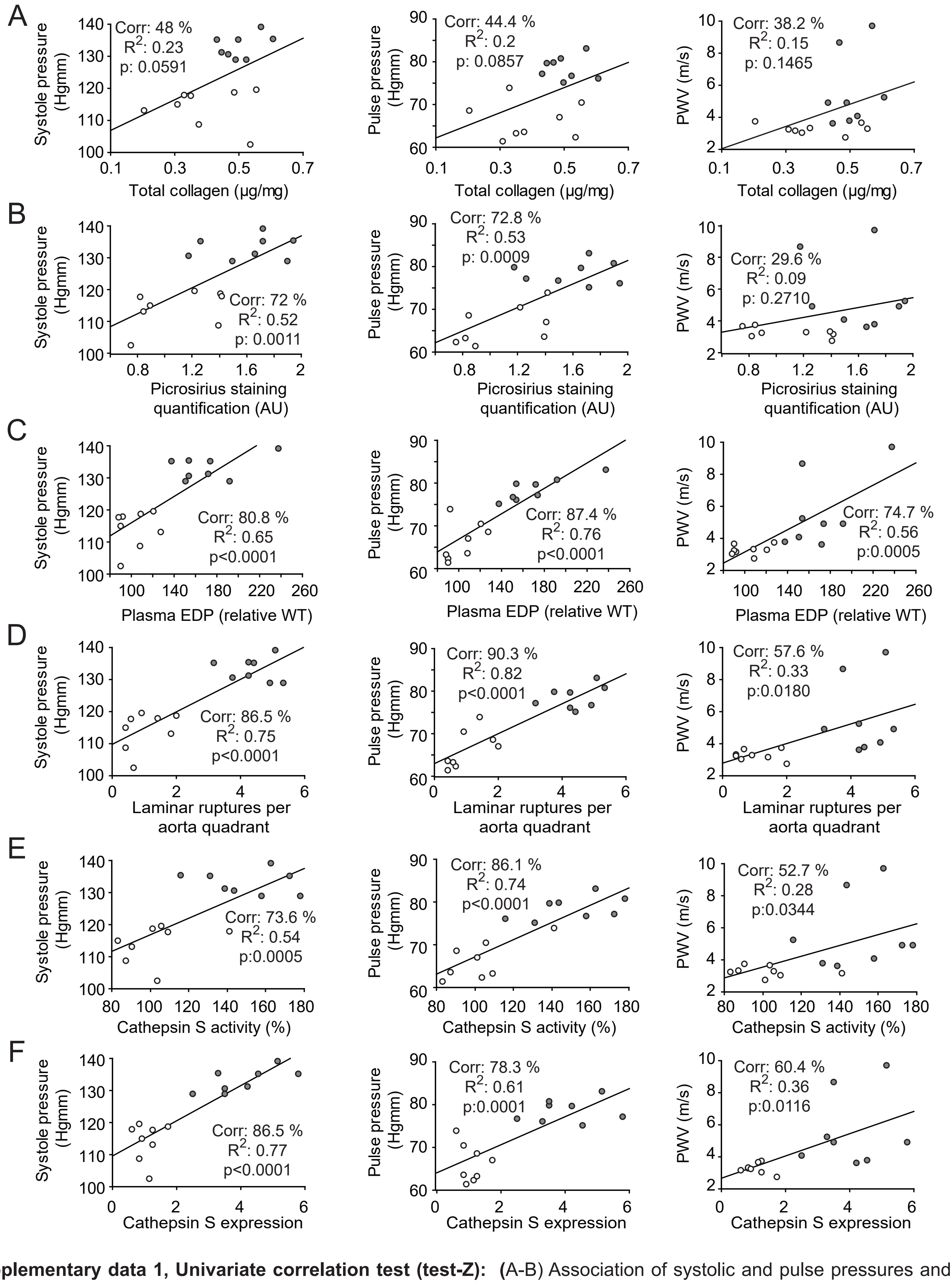

Supplementary data 1, Univariate correlation test (test-Z): (A-B) Association of systolic and pulse pressures and pulse wave velocity, PWV (obtained in figure 2) with total collagen assay (panel A), picrosirius stainning (panel B) obtained figure 3. (C-D) Association of systolic and pulse pressures and pulse wave velocity, PWV with plasma EDP assay (panel C), Laminar ruptures per aorta quadrant (panel D) determined in figure 4. (E-F) Association of systolic and pulse pressures and pulse wave velocity, PWV with cathepsin S activity (panel E) and cathepsin S expression (panel F) determined in figure 5.
